# Genome-Wide Profiling of bZIP Transcription Factors and FocbZIP11’s Impact on Fusarium TR4 Pathogenicity

**DOI:** 10.3390/ijms26041452

**Published:** 2025-02-09

**Authors:** Yanling Xie, Huoqing Huang, Yile Huo, Wenlong Yang, Yuqing Li, Siwen Liu, Chunyu Li

**Affiliations:** 1 Key Laboratory of South Subtropical Fruit Biology and Genetic Resource Utilization, Ministry of Agriculture and Rural Affairs, Guangdong Provincial Key Laboratory of Science and Technology Research on Fruit Tree, Institute of Fruit Tree Research, Guangdong Academy of Agricultural Sciences, Guangzhou 510640, China; xieyanling18@163.com (Y.X.); hqhuang07@163.com (H.H.); lele940910@163.com (Y.H.); syt226huyl@163.com (W.Y.); liyuqing_lee@163.com (Y.L.); 2 Key Laboratory of Tropical Fruit Biology, Ministry of Agriculture and Rural Affairs, Key Laboratory of Hainan Province for Postharvest Physiology and Technology of Tropical Horticultural Products, South Subtropical Crops Research Institute, Chinese Academy of Tropical Agricultural Sciences, Zhanjiang 524091, China; 3 National Key Laboratory for Tropical Crop Breeding, Sanya 572024, China; 4 Guangdong Laboratory for Lingnan Modern Agriculture, Guangzhou 510640, China; 5 Maoming Branch, Guangdong Laboratory for Lingnan Modern Agriculture, Maoming 525000, China

**Keywords:** *Fusarium oxysporum* f. sp. *cubense* tropical race 4, fungal stress adaptation, plant–pathogen interactions, virulence

## Abstract

The basic leucine zipper (bZIP) transcription factor (TF) family performs diverse functions in fungal processes, including vegetative growth, nutrient utilization, stress responses, and invasion. Despite their importance, little is known about the bZIP members in *Fusarium oxysporum* f. sp. *cubense* tropical race 4 (*Foc* TR4), a highly virulent banana pathogen. In this study, we systematically identified 17 bZIPs distributed across 10 *Foc* TR4 chromosomes and classified them into four types based on their protein sequences. Phylogenetic analysis of fungal bZIP TFs revealed that the FocbZIP proteins cluster into 12 groups shared across fungal species. A *cis*-element analysis showed that each *bZIP* promoter contains at least one type of stress response-related element. Furthermore, RNA-seq and RT-qPCR analyses of *FocbZIP* gene expression patterns demonstrated that these genes may serve distinct roles during infection. Notably, the deletion of *FocbZIP11* led to reduced vegetative growth, heightened sensitivity to osmotic, oxidative, and cell wall stresses, and diminished virulence toward banana plantlets. Overall, our findings indicate that *FocbZIP11* plays a critical role in growth, abiotic stress responses, and virulence in *Foc* TR4. This study provides a foundation for the further functional characterization of *FocbZIP* genes, and *FocbZIP11* might serve as a promising target for RNA-based biopesticide control of FWB.

## 1. Introduction

Bananas are a widely cultivated tropical and subtropical fruit with significant economic value, with an annual production of approximately 135.11 million tons [1]. However, Fusarium wilt of banana (FWB), caused by *Fusarium oxysporum* f. sp. *cubense* (*Foc*), is one of the most destructive plant diseases, resulting in severe economic losses globally [2]. Among its variants, *Foc* TR4 infects nearly all banana species and exhibits the highest pathogenicity to Cavendish, the world’s most widely planted cultivar [2]. Among its spores, chlamydospores can survive in soil for many years in the absence of banana hosts, therefore, *Foc* TR4 spreads across Asia, Oceania, Africa, and the Americas quickly [2,3]. Currently, no effective strategies exist to control this pathogen. Thus, identifying and characterizing genes associated with development and virulence in *Foc* TR4 could provide valuable insights for developing targeted approaches to manage FWB.

The basic leucine zipper (bZIP) transcription factors (TFs) represent one of the largest and most widely studied TF families in eukaryotes. bZIP proteins feature two characteristic domains: a highly conserved DNA-binding basic region and a variable leucine zipper region [4]. The basic region comprises approximately 16 amino acids and includes an invariant N-X_7_-R/K-X_9_ motif, which regulates DNA binding and nuclear localization [5]. The leucine zipper region consists of several repeating motifs containing leucine or other bulky hydrophobic amino acids [5,6].

Previous studies have shown that bZIP TFs are involved in development and various stress responses [7]. Many bZIP family members have been identified, with several functionally characterized. In *Magnaporthe oryzae*, 22 bZIP TFs have been identified, seven of which play key roles in pathogenicity, development, stress responses, or metabolism. For example, the endoplasmic reticulum stress response is regulated by *MoHac1*, growth and differentiation are governed by *MoMetR* and its control of amino acid metabolism, appressorium function and invasive hyphal growth is regulated by *MoBzip10*, and nitrogen utilization involves *MoMeaB* [8]. Additionally, *BIP1* is crucial for the expression of early invasion-related genes in appressoria, and *ΔBIP1* mutants are non-pathogenic on rice plants [9].

In *Fusarium graminearum*, 22 *bZIP* genes have been identified [10]. Fgap1 (a homolog of Yap1) mediates oxidative stress responses and trichothecene biosynthesis, while FgTfmI contributes to deoxynivalenol (DON) biosynthesis and the infection process in planta [11,12]. *FpAda1* knockout in *Fusarium pseudograminearum* leads to hyphal growth defects, mycelial branching, and conidia formation, with *ΔFpAda1* mutants showing significantly reduced growth on wheat coleoptiles and barley leaves [13]. Furthermore, *ΔFpkapc* mutants exhibit greater tolerance to ion stress but reduced sensitivity to H_2_O_2_ [14]. These studies underscore the critical roles of *bZIP* genes in regulating growth, development, environmental stress responses, and pathogenicity.

In *Foc*, the *ΔFoatf1* mutant shows significantly reduced tolerance to oxidative stress, which impacts its pathogenicity [15]. Additionally, Ace2 is essential for virulence, cell wall homeostasis, conidiation, and vegetative growth [16]. These findings on atf1 and Ace2 lead us to investigate the potential bZIP family protein biological functions in *Foc* TR4.

Previous studies have investigated the pathogenic mechanism of bZIP TFs in other fungi. Numerous studies have shown that a range of pathogenicity-related gene families were selected to be new target genes for the host-induced gene silencing (HIGS) technique. One of the most extensive target screens using dsRNA treatment was performed on *Sclerotinia sclerotiorum*, 20 of the 59 targets screened significantly reduced disease symptoms [17]. However, to our knowledge, only two bZIP TFs (Foatf1 and FoAce2) have been reported in *Foc* [15,16]. Systematic identification of bZIP TFs and evaluation experiments for HIGS targets have yet to be conducted in *Foc* TR4. This underscores the significant importance of identifying bZIP TFs, which contributes to the discovery of novel fungicide targets for controlling FWB. Thus, a genome-wide identification and characterization of *Foc* TR4 *bZIP* genes (*FocbZIP*s) is crucial for understanding their roles in this pathogen.

We conducted a genome-wide analysis to identify and characterize 17 *bZIP* genes in *Foc* TR4. Specifically, we analyzed gene structures, conserved domains, phylogenetic relationships, chromosomal locations, and expression patterns of *FocbZIP* genes in response to *Foc* TR4 infection. Additionally, we generated *FocbZIP11* mutants and investigated the biological functions of the *FocbZIP11-T1* mutants. This study offers valuable insights into the FocbZIP transcription factors, including the identification of members potentially associated with virulence. By unraveling the molecular functions of bZIP transcription factors in *Foc* TR4, this study lays the foundation for novel interventions against Fusarium wilt, addressing a critical gap in current pathogen management strategies.

## 2. Results

### 2.1. Identification of Foc TR4 bZIP Genes

In total, we identified 17 putative proteins containing the bZIP domain (PF00170), which we designated as *FocbZIP1* to *FocbZIP17*. As summarized in Table 1, the 17 FocbZIP proteins ranged in length from 143 amino acids (FocbZIP17) to 612 amino acids (FocbZIP7). Their theoretical molecular weights spanned from 16.26 kDa (FocbZIP17) to 68.30 kDa (FocbZIP7), while their predicted isoelectric points (pI) ranged from 4.48 (FocbZIP13) to 9.13 (FocbZIP2). Based on predicted subcellular localizations, most FocbZIP proteins were localized in the nucleus, while some, including FocbZIP3, FocbZIP7, and FocbZIP10, were localized in the cytoplasm (Table 1).

### 2.2. Chromosomal Location and Conserved Domain Analysis

**Figure 1:**
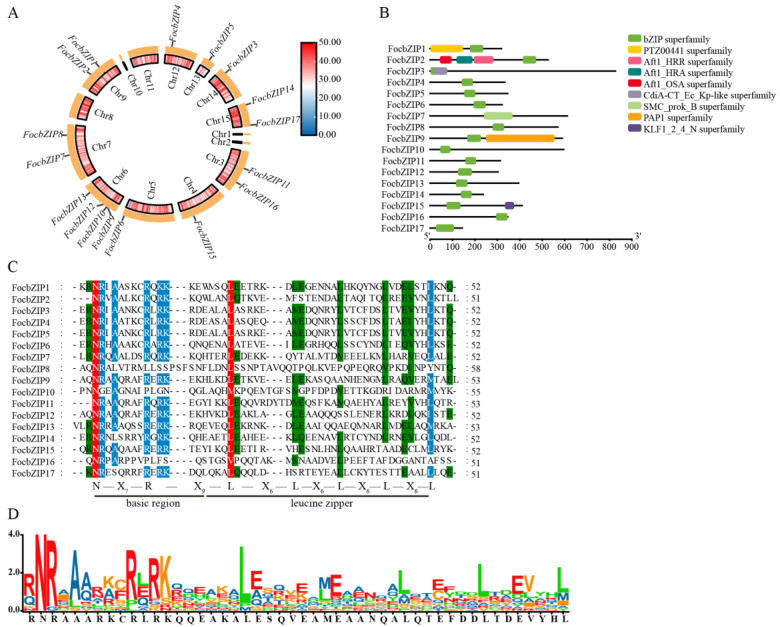
Chromosomal locations and conserved domain distributions of 17 FocbZIP family members in *Foc* TR4. (**A**) Chromosomal locations of *FocbZIP* genes (outer circle) and the gene densities of the chromosomes (inner circle). The color scale represents variations in gene densities, with red signifying an increase and blue indicating a decrease. (**B**) Conserved domains of FocbZIP proteins highlighted in different colors. (**C**) Multiple sequence alignment of the bZIP domain in 17 FocbZIP proteins in *Foc* TR4. Red, blue, and green indicate the conserved percentage of 100%, >80%, and >60%, respectively. The short, black lines at the bottom represent a typical bZIP domain. (**D**) The sequence logo formed by the bZIP domain from the 17 FocbZIP proteins, and the larger letters of the amino acid residues, the more frequently they appear at the same site.

The distribution of the 17 *FocbZIP* genes across the chromosomes is shown in Figure 1A. These genes were spread across 10 chromosomes: Chromosome 4 (Chr4), Chr5, Chr12, Chr13, and Chr14 each contained one *FocbZIP* gene; Chr3, Chr7, Chr9, and Chr15 each contained two *FocbZIP*s; and Chr6 contained four *FocbZIP*s. Although the *FocbZIP* genes were not evenly distributed, no chromosome showed a preferential accumulation of these genes.

Several conserved domains were identified in the 17 FocbZIP proteins, as highlighted in Figure 1B. All FocbZIP proteins contained a typical bZIP domain. Additionally, FocbZIP1 featured the PTZ00441 superfamily domain, and FocbZIP15 contained the KLF1_2_4_N superfamily domain. FocbZIP2 possessed the Atf1-HRA domain (PF11786), which is involved in meiotic recombination [18], while FocbZIP9 contained the PAP1 domain, which regulates antioxidant gene expression in response to H_2_O_2_ [19].

To further explore the domain characteristics of the 17 FocbZIP proteins, we performed a multiple amino acid sequence alignment (Figure 1C,D). Most bZIP domains shared the same N-X_7_-R pattern. However, the core arginine (R) residue in FocbZIP10 and FocbZIP16 is replaced by proline (P), and FocbZIP8 by leucine (L). The first leucine (L) in the leucine zipper region is more conserved than subsequent leucines, except in FocbZIP10 and FocbZIP16, where it is replaced by methionine (M) and valine (V), respectively. The later leucines showed greater variation, often being substituted with other bulky hydrophobic amino acids, such as methionine (M), alanine (A), or serine (S).

### 2.3. Motif, Gene Structure and Phylogenetic Analysis

**Figure 2:**
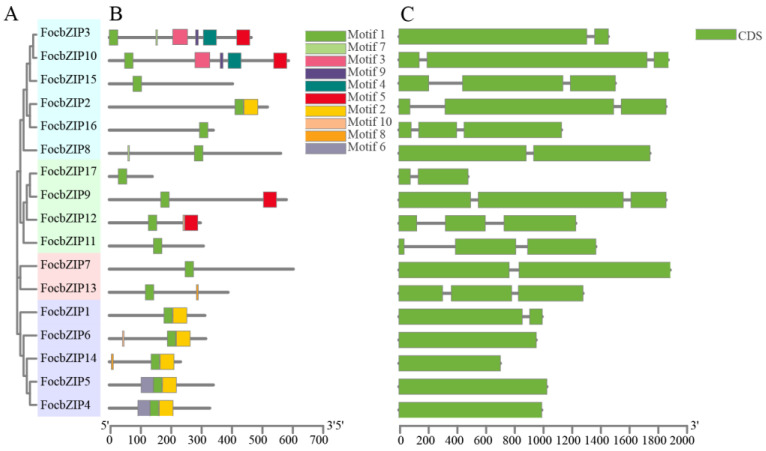
Analysis of phylogenetic tree, conserved motifs and gene structures in 17 bZIP family members. (**A**) Classification of FocbZIP proteins (Groups I–IV) represented in different colors. (**B**) Distribution of motifs within each FocbZIP protein. The gray lines indicate the protein lengths. Ten motifs are highlighted in different colored boxes. (**C**) *FocbZIP* gene structures. Exons are indicated by green boxes and the introns are represented by the black lines between exons.

We constructed a phylogenetic tree based on sequence alignment results, revealing that the 17 FocbZIP proteins are grouped into four distinct categories (I–IV) (Figure 2A). To analyze the structural similarities among FocbZIP proteins, we identified 10 conserved motifs using the MEME 5.5.7 website (Figure 2B). These motifs, ranging from 6 to 50 amino acids in length, were distributed across the 17 FocbZIP proteins, with each protein containing between 1 and 6 motifs. Notably, motif 1 was present in all sequences. Genes clustered within the same group shared similar motif compositions; for instance, all members of the IV subfamily contained motifs 1 and 2. The gene structures are shown in Figure 2C. While some *FocbZIP* genes, such as *FocbZIP4*, *FocbZIP5*, *FocbZIP6*, and *FocbZIP14*, contained only a single exon, others included one or two introns. Specifically, five *FocbZIP* genes harbored one intron, and eight genes contained two introns.

**Figure 3:**
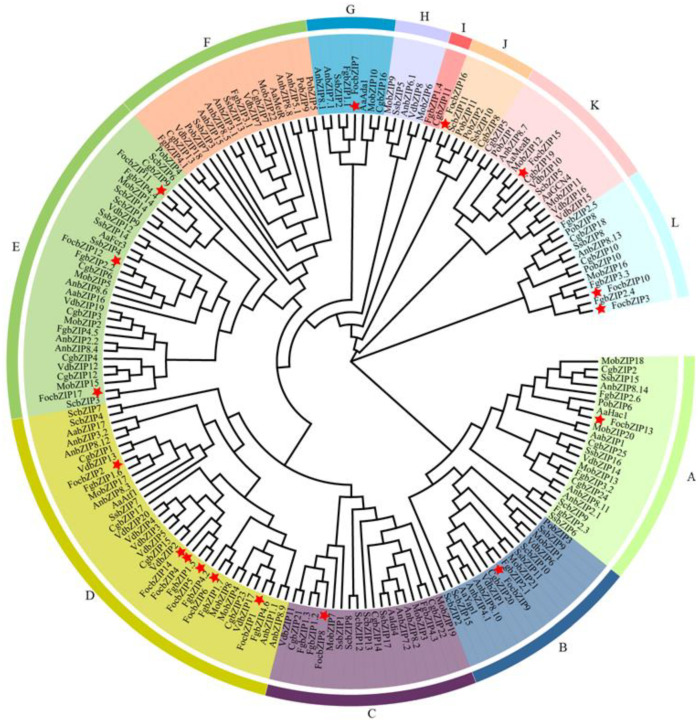
Phylogenetic analysis of bZIP proteins in 10 fungal species. Proteins were clustered into 12 clades (A–L) indicated by different colored lines, FocbZIP proteins are marked with red stars. Aa: *Alternaria alternate*; An: *Aspergillus nidulans*; Cg: *Colletotrichum gloeosporioides*; Foc: *Fusarium oxysporum* f. sp. *cubense* tropical race 4; Fg: *Fusarium graminearum*; Mo: *Magnaporthe oryzae*; Po: *Pleurotus ostreatus*; Sc: *Saccharomyces cerevisiae*; Ss: *Sclerotinia sclerotiorum*; Vd: *Verticillium dahliae*.

To further examine the evolutionary relationships and classification of the FocbZIP family, we performed a phylogenetic analysis of bZIP TFs from 10 fungal species, encompassing a total of 186 bZIP proteins (Figure 3). These proteins were categorized into 12 clades, labeled A through L. The 17 FocbZIP proteins were distributed across nine clades. Of these, nine FocbZIP proteins were grouped into two major clades: FocbZIP1, FocbZIP2, FocbZIP4, FocbZIP5, FocbZIP6, and FocbZIP14 were part of clade D, while FocbZIP11, FocbZIP12, and FocbZIP17 belonged to clade E. The remaining eight FocbZIP proteins were assigned to seven smaller clades. Within clade D, FocbZIP4, FocbZIP5, and FocbZIP14 were closely related, suggesting a recent genome duplication event. Interestingly, clade D lacked bZIPs from *Pleurotus ostreatus*, indicating that bZIP TFs likely diverged later in this species.

### 2.4. cis-Element Analysis

**Figure 4:**
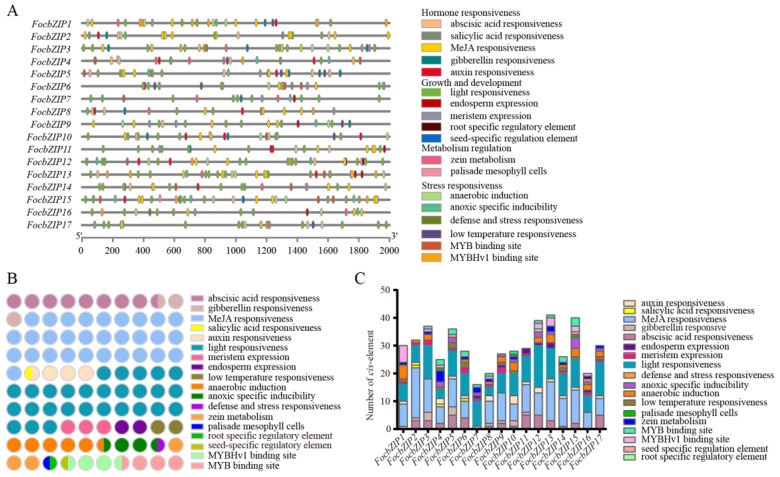
*cis*-element analysis in *FocbZIP* promoters of *Foc* TR4. (**A**) Distribution of different types of *cis*-elements in *FocbZIP*s. (**B**) Number of *cis*-elements related to a specific function. Different functions are represented in different colors and the number of circles indicate the number of functions. (**C**) Number of *cis*-elements contained in each *FocbZIP* promoter. Different functions are represented in different colors.

To investigate the potential roles of *FocbZIP* genes, we analyzed *cis*-elements within 2 Kb upstream of their promoters. In total, 504 *cis*-elements were identified in the promoter regions of the 17 *FocbZIP*s and categorized into four groups (Figure 4A). Among these, hormone-responsive elements were the most abundant, while elements related to metabolism regulation were the least represented (Figure 4B). Light-responsive elements, classified under the growth and development group, were present in all 17 *FocbZIP*s. Each *FocbZIP* gene contained more than 16 *cis*-elements (Figure 4C), with the number ranging from 16 in *FocbZIP7* to 41 in *FocbZIP13*. Notably, 11 *FocbZIP*s contained *cis*-elements that interact with *MYB* genes, which were involved in drought response and flavonoid synthesis. This suggests that *FocbZIP*s may perform biological functions by regulating MYB activity. In summary, the diverse *cis*-elements identified in the *FocbZIP* promoters indicate that these genes likely participate in various signaling pathways.

### 2.5. Expression Patterns of FocbZIP Genes During the Infection Stages

**Figure 5:**
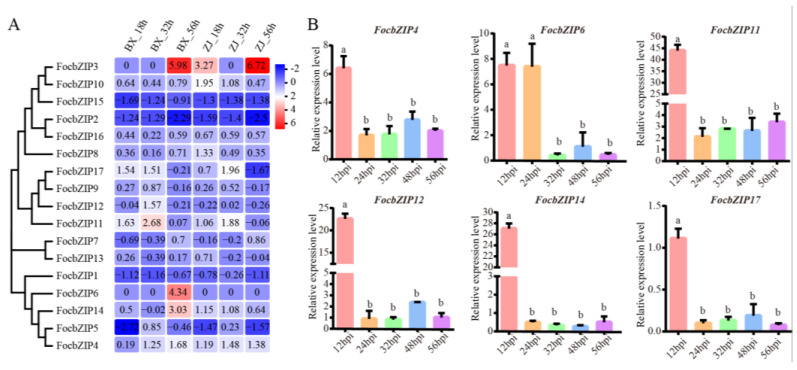
Expression profiles and RT-qPCR gene expression patterns of *FocbZIP*s at early infection stages. (**A**) Heatmap showing the expression data for each *FocbZIP*. Original FPKM values of *FocbZIP* genes were transformed by log2. Red indicates up-regulation and blue indicates down-regulation. (**B**) Expression of six *FocbZIP* genes at different points during early infection processes by *Foc* TR4 in banana roots. Fungal reference gene *FocEF1α* (elongation factor 1-alpha) was used to normalize expression levels. Vertical bars represent means ± SD (*n* = 3). Different letters above the columns indicate the significant differences (via one-way analysis of variance, *p* < 0.05).

To examine whether *FocbZIP* genes are involved in infection processes, we analyzed their expression profiles using RNA-seq data during early infection stages at 18, 32, and 56 h post-inoculation (hpi). As shown in Figure 5A, *FocbZIP*s exhibited diverse expression patterns in the resistant cultivar (ZJ) and the susceptible cultivar (BX). Six *FocbZIP* genes (*FocbZIP4*, *FocbZIP6*, *FocbZIP11*, *FocbZIP12*, *FocbZIP14*, and *FocbZIP17*) showed significantly higher expression in the susceptible cultivar than in the resistant one. We further evaluated the expression levels of these six *FocbZIP* genes in banana roots infected with the *Foc* TR4 strain II5 using RT-qPCR at various time points. All six genes displayed peak expression at 12 hpi, with *FocbZIP11* showing the highest level of upregulation, approximately 44-fold higher compared to other time points (Figure 5B). These findings suggest that *FocbZIP* genes, particularly *FocbZIP11*, play critical roles in the fungal pathogenicity of *Foc* TR4. Based on these results, we selected *FocbZIP11* for further functional analyses.

### 2.6. FocbZIP11 Was Involved in Stress Responses

**Figure 6:**
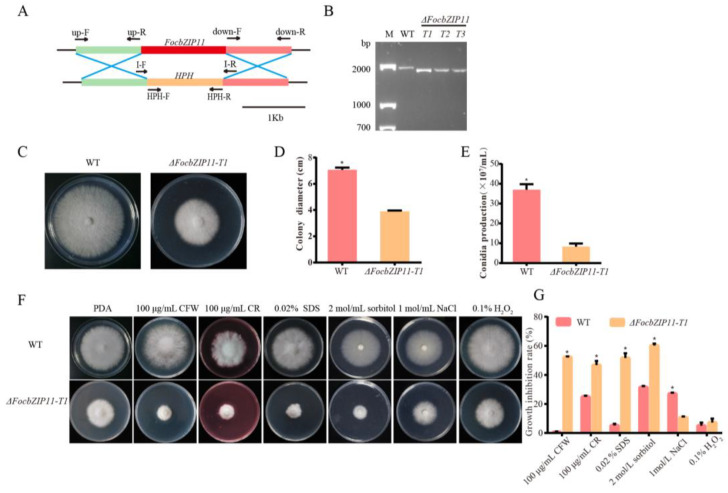
Phenotypic analyses and sensitivity tests of the *ΔFocbZIP11-T1* mutant. (**A**) Schematic diagram of the *FocbZIP11* gene deletion process and primers used to verify the gene replacement event. (**B**) Confirmation of the *ΔFocbZIP11-T1* mutant via PCR amplification. M: 2000 bp marker. A 1790 bp fragment from the *ΔFocbZIP11-T1* mutant containing a partial upstream sequence and downstream sequence of the *FocbZIP11* and *HPH* gene sequences was amplified by the primer pair I-F/I-R. This confirmed that the 1421 bp fragment of the *FocbZIP11* gene was replaced by the 1349 bp fragment of the *HPH* gene. (**C**) Colony morphology of the WT and *ΔFocbZIP11-T1* strains after growth on PDA plates for 6 days. (**D**) Colony diameter of WT and *ΔFocbZIP11-T1* after growth for 6 days. (**E**) Conidia production of the WT and *ΔFocbZIP11-T1* mutants. (**F**) Morphology of the WT and *ΔFocbZIP11-T1* mutants on PDA plates with abiotic stresses (100 μg/mL CFW, 100 μg/mL CR and 0.02% SDS, 2 mol/L sorbitol, 1 mol/L NaCl, 0.1% H_2_O_2_) 6 days after inoculation. (**G**) Quantification of colony size inhibition in each strain. The inhibition rate is calculated as the percentage growth reduction in the treated samples compared to the control. Vertical bars represent means ± SD (*n* = 3). Asterisks indicate significant differences (*p* < 0.05).

To investigate the role of *FocbZIP11* in fungal pathogenicity, we constructed a gene fragment containing a hygromycin resistance gene (*HPH*) cassette and replaced the *FocbZIP11* region in the *Foc* TR4 strain II5 with the *HPH* cassette using polyethylene glycol (PEG) and CaCl_2_ (Figure 6A). *FocbZIP11*-deletion mutants (*ΔFocbZIP11*) were obtained and confirmed by PCR using the I-F/R primer pairs (Table S1, Figure 6B). Three deletion mutants—T1, T2, and T3—exhibited similar phenotypes, with T1 (*ΔFocbZIP11-T1*) selected for further analysis. Compared to the wild type (WT), the *ΔFocbZIP11-T1* mutant showed a 44.8% reduction in colony size on PDA plates (Figure 6C,D). We also assessed conidia production by *ΔFocbZIP11-T1* and WT strains in PDB liquid medium for 3 days, followed by counting conidia using a hemocytometer. The *ΔFocbZIP11-T1* mutant produced significantly fewer conidia than the WT strain (Figure 6E). These results indicate that the *ΔFocbZIP11-T1* mutant exhibits significant differences from the WT in both mycelial morphology and conidiation.

To assess the role of *FocbZIP11* in stress responses, we compared the fungal growth of the WT and *ΔFocbZIP11-T1* strains on PDA plates supplemented with cell wall-targeting agents (CFW, CR, and SDS), osmotic stress agents (NaCl and sorbitol), and the oxidative stress agent H_2_O_2_ (Figure 6F,G). The *ΔFocbZIP11-T1* mutant exhibited increased sensitivity to 100 μg/mL CFW, 100 μg/mL CR, and 0.02% SDS compared to the WT strain, with colony diameters reduced by 52.3%, 46.7%, and 51.6%, respectively. The mycelial growth of the *ΔFocbZIP11-T1* mutant was significantly inhibited on PDA plates containing 2 mol/L sorbitol, with a growth reduction of 59.9%, while the WT strain showed only a 31.5% inhibition. In contrast, the mutant exhibited a 10.6% inhibition on PDA plates with 1 mol/L NaCl, while the WT strain showed a 27.0% inhibition. Additionally, exposure to 0.1% H_2_O_2_ reduced the growth of the *ΔFocbZIP11-T1* mutant by 7.2%, compared to a 5.0% reduction in the WT strain. These results suggest that *FocbZIP11* plays a role in fungal stress responses.

### 2.7. FocbZIP11 Contributes to Foc TR4 Virulence

**Figure 7:**
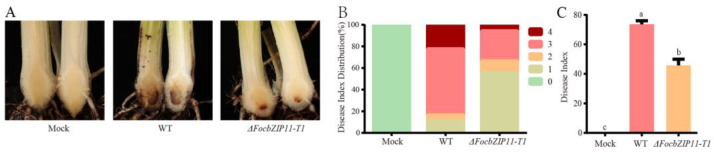
Pathogenicity assay of *ΔFocbZIP11-T1* mutants. (**A**) Disease symptoms, (**B**) distribution of plant disease scores, and (**C**) disease indices in banana plantlets with roots inoculated with the WT and *ΔFocbZIP11-T1* mutant strains 40 days post-inoculation. Different letters above the columns indicate significant differences (one-way analysis of variance, *p* < 0.05). Data are presented as means ± SDs from three independent experiments.

To further investigate whether the *FocbZIP11* gene is required for the virulence of *Foc* TR4, we inoculated the susceptible cultivar BX with the WT and *ΔFocbZIP11-T1* strains. The water-inoculated banana plants (mock) showed no internal symptoms of *Foc* TR4 during a 40-day observation period (Figure 7A). In contrast, plants inoculated with the WT strain developed significant browning in their corms, with a disease incidence of 100% and a disease index of 73.6% after 40 days (Figure 7A–C). In comparison, more than 66.6% of plants inoculated with the *ΔFocbZIP11-T1* mutant exhibited only mild disease symptoms after 40 days, with a disease index of 45.8% (Figure 7A–C). These results indicate that the virulence of the *ΔFocbZIP11-T1* mutant was significantly reduced. Overall, our findings suggest that *FocbZIP11* plays a crucial role in the virulence of *Foc* TR4, as its deletion impairs pathogenicity.

## 3. Discussion

The bZIP TF family plays a critical role in various biological processes in fungi, including growth, stress responses, nutrient utilization, and infection [7,8]. Previous studies have shown that bZIP TF Atf1 regulates pathogenesis by modulating oxidative stress, while Ace2 controls virulence, growth, and cell wall homeostasis in *Foc* [15,16]. However, a comprehensive genome-wide analysis of the *bZIP* gene family in *Foc* TR4 has not been conducted. Therefore, we performed an in-depth analysis of the *bZIP* gene family in *Foc* TR4 and functionally characterized the roles of *FocbZIP11* in fungal growth, stress responses, and pathogenicity.

In this study, we identified and systematically analyzed 17 *bZIP* genes in *Foc* TR4. The number of bZIP TFs in *Foc* TR4 is lower than in other fungi, such as *Magnaporthe oryzae* (22), *Ustilaginoidea virens* (28), *Fusarium graminearum* (26), and *Fusarium fujikuroi* (44), suggesting that the reduced number of *bZIP* genes in *Foc* TR4 is a result of evolutionary divergence compared to other fungi [10,20,21,22]. The 17 *bZIP* genes were unevenly distributed across 10 chromosomes, with chromosome 6 containing the most genes (*FocbZIP9*, *FocbZIP10*, *FocbZIP12*, *FocbZIP13*). These genes, despite being located on the same chromosome, belong to different clades, reflecting their involvement in diverse functions [23]. For example, *FocbZIP9*, *FocbZIP10*, *FocbZIP12*, and *FocbZIP13* are each classified into distinct clades. Phylogenetic analysis of FocbZIP proteins, along with selected fungal bZIP TFs, revealed that the FocbZIP proteins are grouped into nine clades, representing 75% of the total clades. This suggests that these proteins predate the divergence of these fungal species. Additionally, four genes (*FocbZIP4*, *FocbZIP5*, *FocbZIP6*, and *FocbZIP14*), which are clustered in clade D and group IV, share identical motifs (motif 1 and motif 2) and possess a single exon, indicating that FocbZIP proteins have a conserved structure. Most of the potential bZIP domains also maintain the conserved residue pattern (N-X_7_-R), similar to findings in *Coniothyrium minitans*, *Cytospora chrysosperma*, *Fusarium graminearum*, and *Pleurotus ostreatus* [7,10,24,25].

bZIP proteins play key roles in various stress responses, including drought, low temperature, anaerobic conditions, and pathogen defense [26,27,28]. A *cis*-element analysis of the *FocbZIP* promoter regions revealed associations with growth and development, hormone responsiveness, stress responses, and metabolic regulation. These findings suggest that bZIP proteins may be regulated by a range of stress-related transcription factors, thereby contributing to stress response regulation.

In *Magnaporthe oryzae*, several *bZIP* genes, including *MoATF1*, *MoHAC1*, *MoAP1*, *MoBZIP10*, and *MoMETR*, are essential for host infection. The expression of these genes is significantly up-regulated during infection. Notably, *BIP1*, a novel bZIP transcription factor, binds to a GCN4-like TGACTC motif. The *BIP1* binding site in the promoter of MGG_08381 is critical for appressorium-specific expression in *Magnaporthe oryzae* [8,9,19]. Additionally, in *Fusarium oxysporum* f.sp. *cubense*, three beauvericin (BEA) biosynthesis genes are significantly down-regulated in *ΔFoAce2* mutants. *FoAce2*-binding motifs have been identified in the promoters of these genes, suggesting that BEA biosynthesis is regulated by *FoAce2*, which contributes to reduced virulence against banana hosts [16]. These findings underscore the important role of bZIP transcription factors in infection processes. Our transcriptome analysis and RT-qPCR results further support the idea that several *bZIP* genes are significantly altered at the early stages of *Foc* TR4 infection, highlighting their critical roles during the infection process. For example, *FocbZIP11* showed the highest level of upregulation compared to other time points, so we selected *FocbZIP11* for further functional analyses.

Numerous studies have demonstrated that *bZIP* genes play crucial roles in vegetative growth, cell development, pathogenicity, and responses to abiotic stress. For instance, in *Fusarium pseudograminearum*, the *ΔFpAda1* mutant exhibited defects in hyphal growth, mycelial branching, and conidia formation [13]. In *Coniothyrium minitans*, *ΔCmbZIP16* mutants showed hypersensitivity to oxidative stress [7]. In *Alternaria alternata*, knockout of 12 bZIP transcription factors led to reduced growth of *ΔAaAtf1* under copper stress, diminished aerial hyphal growth in *ΔAaHac1* and *ΔAaGcn4* mutants, and hypersensitivity to oxidative stress in *ΔAaYap1*, suggesting that bZIP TFs are involved in growth and stress responses [29].

In this study, the *ΔFocbZIP11* mutant exhibited significantly reduced vegetative growth and conidia production, similar to the *ΔFpAda1* mutant. Furthermore, *ΔFocbZIP11-T1* displayed heightened sensitivity to osmotic stress, oxidative stress, and cell wall stress, resembling the phenotypes observed in *ΔCmbZIP16* and *ΔAaYap1* mutants. These findings suggest that *FocbZIP11* plays a critical role in the subtle regulation of various biological processes. Further functional studies on *FocbZIP11* are necessary to gain a deeper understanding of its mechanisms and roles.

Previous studies have highlighted the involvement of many *bZIP* genes in fungal virulence. For example, in *Cytospora chrysosperma*, *CcbZIP05* and *CcbZIP23* are essential for fungal growth, virulence, and regulation of chitin synthesis-related genes [24]. In *Alternaria alternata*, *AaHac1* is critical for virulence on citrus leaves [29]. In *Fusarium fujikuroi*, *FfZIP2*, *FfZIP5,* and *FfZIP10* are indispensable for pathogenesis [22]. In the present study, we found that *FocbZIP11* is involved in the virulence of *Foc* TR4. The *ΔFocbZIP11-T1* mutant exhibited reduced virulence on banana roots, a result similar to that observed in the *ΔFoAce2* mutant. This suggests that *FocbZIP11* may interact with motifs in virulence-associated genes.

Overall, *FocbZIP11* plays critical roles in fungal growth, stress response, and pathogenicity. We hypothesized that other *bZIP* genes may perform similar functions in *Foc* TR4; however, these *bZIP* genes have not been characterized in the present study. In future studies, we intend to perform knockout experiments on these *bZIP* genes in order to investigate potential functions. Previous studies in our group have indicated that ergosterol synthesis genes (*ERG6* and *ERG11*) are crucial roles for conidial germination in *Foc* TR4. Additionally, two transgenic bananas lines (ERG6-RNAi and ERG11-RNAi), generated using HIGS technology, significantly enhance the resistance of Cavendish bananas to *Foc* TR4 [30,31]. Results from the studies of FocbZIP11 will provide new potential targets for the development of sustainable control strategies against FWB and for breeding disease-resistance banana varieties utilizing HIGS technology. In future studies, we plan to suppress the *FocbZIP11* gene to control FWB using the HIGS technique or by applying its dsRNAs.

In summary, we identified bZIP TFs across the *Foc* TR4 genome and analyzed their intron arrangements and expression patterns during infection. Our findings indicate that several *FocbZIP* genes are involved in the infection process. Notably, *FocbZIP11* plays a significant role in vegetative growth, conidiation, stress responses, and pathogenicity of *Foc* TR4. These results offer valuable insights into the FocbZIP TF family and provide a foundation for further investigation into the regulatory mechanisms of bZIP TFs in *Foc* TR4.

## 4. Materials and Methods

### 4.1. Plant Material and Fungal Strains

Banana plantlets (*Musa* AAA Cavendish subgroup, cv. Brazil) with five to six leaves were cultivated in a greenhouse at 28 °C under a 14/10 h light/dark cycle. The WT strain, *Foc* TR4 strain II5 (NRRL#54006), was used in this study. Both the WT and mutant strains were cultured on potato dextrose agar (PDA) plates to assess vegetative growth. To produce conidia, the WT and mutant strains were cultured in potato dextrose broth (PDB) at 28 °C on a shaker at 180 rpm for 3 days. The conidial concentration in the suspensions was determined using a hemacytometer [32].

Stress sensitivity assays were conducted on PDA plates supplemented with 100 μg/mL calcofluor white (CFW), 100 μg/mL congo red (CR), 0.02% sodium dodecyl sulfate (SDS), 2 mol/L sorbitol, 1 mol/L NaCl, or 0.1% H_2_O_2_ (Sangon Biotech Co., Ltd., Shanghai, China). After incubation for 6 days at 28 °C in the dark, colony diameters were measured to calculate the growth rate [32,33,34,35].

### 4.2. Identification of bZIP Transcription Factors in Foc TR4

The complete genome sequence and annotated file of *Foc* TR4 (GCA_007994515) were downloaded from the Ensemble database (https://fungi.ensembl.org/index.html, accessed on 11 September 2024). The profile Hidden Markov Model (HMM) of the bZIP domain (PF00170) was retrieved from the Pfam 37.2 database [36]. bZIP TFs were identified using HMMER 3.0 and TBtools 2.142 software [37,38]. These proteins were further validated through the online Conserved Domain Database (CDD 3.20, https://www.ncbi.nlm.nih.gov/Structure/bwrpsb/bwrpsb.cgi, accessed on 15 September 2024) and SMART 9 (http://smart.embl-heidelberg.de/, accessed on 15 September 2024) [39,40]. The ExPASy 3.0 server (https://web.expasy.org/protparam/, accessed on 15 September 2024) was used to analyze the physiochemical properties of bZIP proteins, including amino acid length, molecular weight (MW), and isoelectric point (pI) [41]. Subcellular localization was predicted using BaCelLo (https://busca.biocomp.unibo.it/bacello/, accessed on 15 September 2024), as detailed in Table 1 [42].

### 4.3. Gene Structural Characterization and Phylogenetic Analysis

The presence of typical bZIP domains in predicted protein sequences was confirmed using the CDD 3.20 (https://www.ncbi.nlm.nih.gov/Structure/bwrpsb/bwrpsb.cgi, accessed on 15 September 2024) and Multiple Em for Motif Elicitation 5.5.7 (MEME) (https://meme-suite.org/meme/tools/meme, accessed on 15 September 2024) with an E-value threshold of 0.01 [40,43]. Chromosomal, intron, and exon information were obtained from the genome annotation file. All figures were generated using TBtools 2.142 software [38].

We aligned the amino acid sequences of the 17 identified bZIP domains using MEGA 6, with minor adjustments made using GeneDoc 2.7 [44,45]. TBtools 2.142 software was then used to highlight the consensus sequence for the bZIP domains [38]. We performed multiple sequence alignment of fungal bZIP TFs from *Fusarium graminearum* (Fg), *Aspergillus nidulans* (An), *Magnaporthe oryzae* (Mo), *Pleurotus ostreatus* (Po), *Sclerotinia sclerotiorum* (Ss), *Alternaria alternate* (Aa), *Saccharomyces cerevisiae* (Sc), *Colletotrichum gloeosporioides* (Cg), and *Verticillium dahliae* (Vd) using MUSCLE 3.8.1551 (https://www.ebi.ac.uk/jdispatcher/msa/muscle?stype=protein, accessed on 16 September 2024) [10,25,29]. The resulting phylogenetic tree was visualized using Evolview version 3 (https://www.evolgenius.info/evolview/#/treeview, accessed on 16 September 2024) [46].

TBtools 2.142 software was used to extract the 2 Kb upstream promoter regions of the *FocbZIP* genes. These sequences were submitted to the PlantCARE 17 tool (http://bioinformatics.psb.ugent.be/webtools/plantcare/html/, accessed on 21 September 2024) for *cis*-regulatory element prediction. The figure displaying the *cis*-regulatory elements was constructed using TBtools 2.142 software and GraphPad Prism 6.01 software (GraphPad Software, San Diego, CA, USA) [38,47].

### 4.4. Total RNA Extraction and Expression Analysis of FocbZIP Genes

RNA extraction and quantitative real-time reverse transcription polymerase chain reaction (RT-qPCR) methods followed those previously described [35]. Briefly, we extracted total RNA from banana roots using an RNA extraction kit (Accurate Biotech Co., Ltd., Changsha, China) according to the manufacturer’s instructions. Genomic DNA was removed, and reverse transcription was performed using the SuperMix for qPCR (+gDNA wiper) kit (Vazyme Biotech Co., Ltd., Nanjing, China). RT-qPCR was conducted on a CFX Connect™ Real-Time System (BioRad, Hercules, CA, USA) with a SYBR qPCR Master Mix kit (Vazyme Biotech Co., Ltd., Nanjing, China). We used *FocEF1α* (elongation factor 1-alpha) as the reference gene, and each treatment was performed in triplicate, with three technical replicates per experiment. The relative expression of each gene under different conditions was calculated using the 2^−△△CT^ method. The primers used for RT-qPCR are listed in Table S1.

### 4.5. Transcriptome Analysis

We determined the expression patterns of the *FocbZIP* genes at different infection stages by RNA sequencing (RNA-seq). Banana roots were collected and immediately frozen in liquid nitrogen, with three biological replicates per sample. Total RNA was extracted from each sample using RNA extraction kit (Accurate Biotech Co., Ltd., Changsha, China) according to the manufacturer’s instructions. A Bioanalyzer 2100 system (Agilent Technologies, Santa Clara, CA, USA) was used for quality analysis. RNA-Seq libraries were prepared with the TruSeq RNA Sample preparation kit (Illumina Inc., San Diego, CA, USA) according to the manufacturer’s instructions and were sequenced using the Illumina HiSeq ×10 (Illumina Inc., San Diego, CA, USA) to generate 150 bp paired-end reads. Removing the dirty raw reads were using Trimmomatic 0.36 [48]. We aligned the filtered reads to the *Foc* TR4 genome using Hisat2 2.0.5 [49]. Differential gene expressions were analyzed using the DESeq2 1.42.0 R package 4.3.1 [50]. Genes with an FPKM (Fragments per kilobase of transcript per million fragments mapped) greater than a two-fold change (*p* < 0.05) were considered differentially expressed. We visualized the heatmap using Evolview version 3 (https://www.evolgenius.info/evolview/#/treeview, accessed on 16 September 2024) [46]. Some genes were selected for further confirmation by RT-qPCR following the procedure described above.

### 4.6. Construction of the Gene Deletion Mutant for FocbZIP11

We constructed deletion mutants of *FocbZIP11* using the protocol described previously [51]. Briefly, we amplified 1 Kb of the 5′- and 3′-flanking sequences of *FocbZIP11* using two pairs of primers (up-F/R and down-F/R) (Table S1), with genomic DNA from strain II5 as the template and 2× Phanta Max Master Mix (Vazyme Biotech Co., Ltd., Nanjing, China). And we also amplified sequences of hygromycin resistance gene cassette (*HPH*) using primers (HPH-F/HPH-R) (Table S1), with 2× Phanta Max Master Mix (Vazyme Biotech Co., Ltd., Nanjing, China). The upstream and downstream fragments were then ligated with a hygromycin resistance gene fragment by a primer-free PCR reaction. Using the resulting PCR products as the template, we performed another PCR with primers up-F/down-R. The 1421 bp fragment of the *FocbZIP11* gene was replaced by a hygromycin-resistance cassette, driven by the TrpC promoter, which was amplified from the pBS-HPH1 vector [52]. We transformed the PCR products into protoplasts of strain II5 using a PEG-mediated method. The deletion transformants were selected on PDA plates containing hygromycin B (50 μg/mL, Sangon Biotech Co., Ltd., Shanghai, China) and cultured at 28 °C for 6 days. We identified the mutants by PCR using primers I-F/R (Table S1).

### 4.7. Virulence Assays

We selected banana plantlets at the five- to six-leaf stage for pathogenicity testing, following the method described previously [53]. Conidia from the WT and *ΔFocbZIP11-T1* mutant strains were collected after 5 days of culturing in PDB. The conidial suspension was adjusted to 1 × 10^7^ conidia/mL for banana root inoculation by irrigation. We soaked the banana plantlet roots in the suspension for 30 min, replanted the plantlets in pots, and placed them in a greenhouse at 28 °C. The experiment was repeated three times, with 30 plantlets per treatment. We evaluated disease symptoms 40 days after inoculation. The disease index was scored as follows: 0 (no symptoms), 1 (some brown spots on the inner rhizome), 2 (browning of less than 1/4 of the inner rhizome), 3 (browning of up to 3/4 of the inner rhizome), and 4 (dark brown and death of the entire inner rhizome and pseudostem).

### 4.8. Statistical Analysis

We performed statistical analyses using GraphPad Prism 6.01 software (GraphPad Software, San Diego, CA, USA). Statistically significant differences between the mutant and WT strains were determined using a *t*-test (*p* < 0.05) or Tukey’s test (*p* < 0.05). The figures were generated using GraphPad Prism 6.01 software (GraphPad Software, San Diego, CA, USA).

## Figures and Tables

**Table 1:** Genes encoding *FocbZIP* transcription factor.

Gene Name	GenBank	gDNA (bp)	cDNA (bp)	Intron	Protein Length (aa)	MW (kDa)	pI	Subcellular Location
*FocbZIP1*	TXB98385.1	1008	957	1	318	35.69	6.12	Nucleus
*FocbZIP2*	TXC02052.1	1878	1581	2	526	55.32	9.13	Nucleus
*FocbZIP3*	TXB97718.1	1473	1419	1	472	53.34	4.89	Cytoplasm
*FocbZIP4*	TXC00663.1	1005	1005	0	334	37.03	6.76	Nucleus
*FocbZIP5*	TXB97812.1	1041	1041	0	346	38.71	7.00	Nucleus
*FocbZIP6*	TXC07756.1	966	966	0	321	35.89	5.65	Nucleus
*FocbZIP7*	TXC03976.1	1907	1839	1	612	68.30	5.09	Cytoplasm
*FocbZIP8*	TXC05006.1	1765	1713	1	570	62.89	4.52	Nucleus
*FocbZIP9*	TXC05793.1	1878	1770	2	589	63.90	4.88	Nucleus
*FocbZIP10*	TXC11600.1	1894	1791	2	596	65.89	5.33	Cytoplasm
*FocbZIP11*	TXC11556.1	1385	942	2	313	33.64	6.71	Nucleus
*FocbZIP12*	TXC05920.1	1244	912	2	303	33.21	5.66	Nucleus
*FocbZIP13*	TXC06889.1	1295	1188	2	395	43.35	4.48	Nucleus
*FocbZIP14*	TXB96322.1	714	714	0	237	26.62	5.24	Nucleus
*FocbZIP15*	TXC09002.1	1522	1233	2	410	45.35	6.66	Nucleus
*FocbZIP16*	TXC11494.1	1144	1041	2	346	37.24	6.51	Nucleus
*FocbZIP17*	TXB95436.1	487	432	1	143	16.26	7.79	Nucleus

## Data Availability

Data are contained within the article or Supplementary Files.

## Supplementary Materials

The following supporting information can be downloaded at: https://www.mdpi.com/article/10.3390/ijms26041452/s1.
